# Supplementary material for: The Evolution and Characterization of the RNA Interference Pathways in Lophotrochozoa
Source: Genome Biol Evol. 2024 May 7;16(5):evae098. doi: 10.1093/gbe/evae098 (PMC11114477; doi:10.1093/gbe/evae098)
Supplement: evae098_Supplementary_Data [file evae098_supplementary_data.zip › Supplementary_materials.zip!/selected_miRNAs/NoGe_selected_miRNAs/mirdeep_pdf_files/NMRB01000426.1_Notospermus_geniculatus_strain_Ushimado_scaffold426,_whole_genome_shotgun_sequence_13046.pdf]

Provisional ID : NMRB01000426.1_Notospermus_geniculatus_strain_Ushimado_scaffold426,_whole_genome_shotgun_sequence_13046
Score total : 35467.8
Score for star read(s) : 3.9
Score for read counts : 35459
Score for mfe : 1.9
Score for randfold : 0
Score for cons. seed : 3
Total read count : 69563
Mature read count : 69456
Loop read count : 0
Star read count : 107

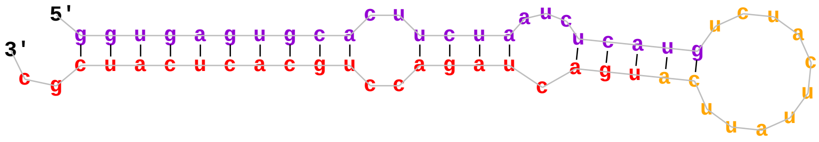

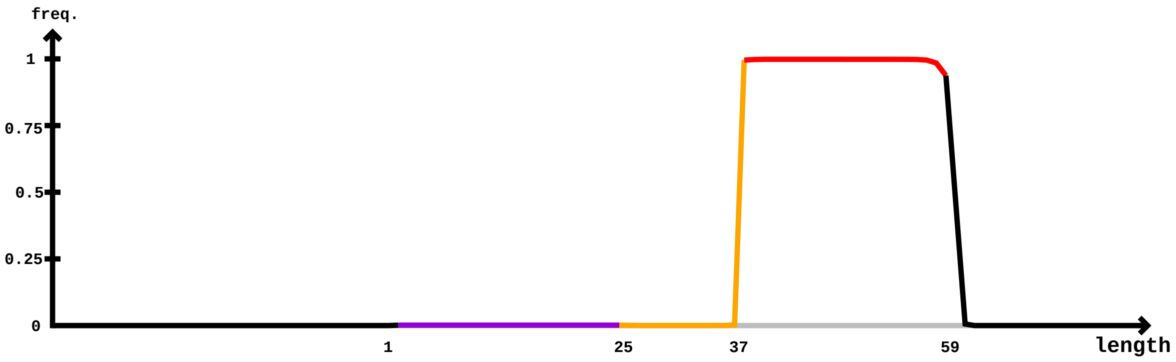

Star Mature

```
5'- uucuguucgugucgcuuauucagauguuuccugcggugagugcacuucuaaucucaugucuacuuauucaugacuagaccugcacucaucgcuggcgacaucuaacccugca  -3'  obs
    uucuguucgugucgcuuauucagauguuuccugcggugagugcacuucuaaucucaugucuacuuauucaugacuagaccugcacucaucgcuggcgacaucuaacccugca       exp
    .............((......(((((((.((.((((((((((((..((((...(((((..........))))).))))..)))))))))))).)).)))))))......)).   reads  mm  sample
    ..................................ggugagugcacuucuaaucucaug......................................................   7      0   Mm3
    ..........................................................ucuacuuauucaugacuagaccugc.............................   1      0   Mm3
    ..........................................................ucuacuuauucaugacuagaccugcacu..........................   1      0   Mm3
    ....................................................................caugacuagaccugcacucaucg.....................   1      0   Mm3
    .....................................................................Cugacuagaccugcacucaucg.....................   1      1   Mm3
    .....................................................................augacuagaccugcacucaucgc....................   1      0   Mm3
    .....................................................................Cugacuagaccugcacucaucgc....................   13     1   Mm3
    .....................................................................Cugacuagaccugcacucaucgcu...................   16     1   Mm3
    ......................................................................ugacuagaccugcacucU........................   2      1   Mm3
    ......................................................................ugacuagaccugcacuca........................   8      0   Mm3
    ......................................................................ugacuagaccugcacucau.......................   14     0   Mm3
    ......................................................................ugacuagaccugcacuUau.......................   1      1   Mm3
    ......................................................................ugacuagaccugcacucCuc......................   5      1   Mm3
    ......................................................................ugacuagaccugcacucauc......................   104    0   Mm3
    ......................................................................ugacuagaccugcacucauU......................   13     1   Mm3
    ......................................................................Ngacuagaccugcacucauc......................   1      1   Mm3
    ......................................................................ugacuagaccugcacucauA......................   1      1   Mm3
    ......................................................................ugacuagaccugcacucGuc......................   1      1   Mm3
    ......................................................................ugacuagaccugcacucaucA.....................   1      1   Mm3
    ......................................................................ugacuagaccugcacucauUg.....................   1      1   Mm3
    ......................................................................ugacuagaccugcGcucaucg.....................   1      1   Mm3
    ......................................................................ugacuagaccugcacucGucg.....................   1      1   Mm3
    ......................................................................ugacuagaccugcacucUucg.....................   1      1   Mm3
    ......................................................................ugacuagaccugcacucaucg.....................   220    0   Mm3
    ......................................................................ugacuagaccugcacucaucU.....................   8      1   Mm3
    ......................................................................ugGcuagaccugcacucaucg.....................   1      1   Mm3
    ......................................................................ugacuagaccugcacuAaucg.....................   1      1   Mm3
    ......................................................................ugacuagaccugcacucaucgU....................   64     1   Mm3
    ......................................................................ugacuagaccugcacucUucgc....................   12     1   Mm3
    ......................................................................ugacuagaccugcacucauUgc....................   2      1   Mm3
    ......................................................................ugacuagacUugcacucaucgc....................   3      1   Mm3
    ......................................................................ugacuagaccugcacucaucgc....................   4590   0   Mm3
    ......................................................................Ngacuagaccugcacucaucgc....................   2      1   Mm3
```

```
                                  Star                                  Mature
uucuguucgugucgcuuauucagauguuuccugcggugagugcacuucuaaucucaugucuacuuauucaugacuagaccugcacucaucgcuggcgacaucuaacccugca
......................................................................ugacuagaccugcacuUaucgc....................   10    1   Mm3
......................................................................ugacuagaccCgcacucaucgc....................   2     1   Mm3
......................................................................ugacuagaccugcaAucaucgc....................   1     1   Mm3
......................................................................ugacuagaccugcacucGucgc....................   23    1   Mm3
......................................................................ugacuaUaccugcacucaucgc....................   1     1   Mm3
......................................................................ugacuagaccugcacucCucgc....................   17    1   Mm3
......................................................................ugacuagaccugcacucaucAc....................   2     1   Mm3
......................................................................ugUcuagaccugcacucaucgc....................   2     1   Mm3
......................................................................ugacuagaccugUacucaucgc....................   1     1   Mm3
......................................................................ugGcuagaccugcacucaucgc....................   2     1   Mm3
......................................................................ugacuagaccugcGcucaucgc....................   3     1   Mm3
......................................................................ugacuagaccugcacucaucUc....................   1     1   Mm3
......................................................................ugacuagUccugcacucaucgc....................   2     1   Mm3
......................................................................ugacuagaccugcacuGaucgc....................   9     1   Mm3
......................................................................ugacuagaccugcacAcaucgc....................   1     1   Mm3
......................................................................ugacuNgaccugcacucaucgc....................   1     1   Mm3
......................................................................ugacuagaccugcacucaCcgc....................   1     1   Mm3
......................................................................ugacuagaccugcaUucaucgc....................   1     1   Mm3
......................................................................ugacuagGccugcacucaucgc....................   2     1   Mm3
......................................................................ugacuagaccugcacuAaucgc....................   22    1   Mm3
......................................................................ugacuagaUcugcacucaucgc....................   1     1   Mm3
......................................................................Agacuagaccugcacucaucgc....................   1     1   Mm3
......................................................................uUacuagaccugcacucaucgc....................   2     1   Mm3
......................................................................ugacuagaccugcacucaucgG....................   3     1   Mm3
......................................................................ugCcuagaccugcacucaucgc....................   1     1   Mm3
......................................................................ugacCagaccugcacucaucgc....................   2     1   Mm3
......................................................................ugacuagaccugcacucauAgc....................   1     1   Mm3
......................................................................ugacuagaccugcUcucaucgc....................   2     1   Mm3
......................................................................ugacuagaccugcacucaucgA....................   13    1   Mm3
......................................................................ugacuagaccGgcacucaucgc....................   6     1   Mm3
......................................................................ugacuagaccAgcacucaucgc....................   8     1   Mm3
......................................................................ugacuagaccugcacucCucgcu...................   9     1   Mm3
......................................................................ugacuagaUcugcacucaucgcu...................   5     1   Mm3
......................................................................ugacuagaccugcacucaAcgcu...................   1     1   Mm3
......................................................................ugacuagGccugcacucaucgcu...................   2     1   Mm3
......................................................................ugacCagaccugcacucaucgcu...................   2     1   Mm3
......................................................................ugacuUgaccugcacucaucgcu...................   1     1   Mm3
......................................................................ugacuagaccAgcacucaucgcu...................   10    1   Mm3
......................................................................ugacuagaccugcacucaucAcu...................   1     1   Mm3
......................................................................ugacuagaccugcacucaucgcC...................   45    1   Mm3
......................................................................ugacuagaccCgcacucaucgcu...................   1     1   Mm3
......................................................................ugacuagaccugcacucaucgcu...................   4828  0   Mm3
......................................................................ugacuagaccugcacucUucgcu...................   20    1   Mm3
......................................................................uAacuagaccugcacucaucgcu...................   2     1   Mm3
......................................................................ugacuagaccGgcacucaucgcu...................   3     1   Mm3
......................................................................ugacuagaccugcacucaucgcG...................   204   1   Mm3
......................................................................ugacuGgaccugcacucaucgcu...................   4     1   Mm3
......................................................................ugGcuagaccugcacucaucgcu...................   2     1   Mm3
......................................................................ugacuagaccugcacuAaucgcu...................   22    1   Mm3
......................................................................ugacuagaccugcacucauAgcu...................   1     1   Mm3
......................................................................ugacuagaccugcacucauUgcu...................   1     1   Mm3
......................................................................ugacuaUaccugcacucaucgcu...................   1     1   Mm3
......................................................................Cgacuagaccugcacucaucgcu...................   3     1   Mm3
......................................................................ugacuagaccugUacucaucgcu...................   1     1   Mm3
......................................................................ugacuagaccugGacucaucgcu...................   1     1   Mm3
......................................................................ugacuagaccugcacuGaucgcu...................   4     1   Mm3
......................................................................ugacuagaccugcacucGucgcu...................   22    1   Mm3
......................................................................ugacuagaccugcacucaucgAu...................   1     1   Mm3
......................................................................ugaUuagaccugcacucaucgcu...................   1     1   Mm3
......................................................................ugacuagaccugcacucaucgUu...................   33    1   Mm3
......................................................................ugacuagUccugcacucaucgcu...................   2     1   Mm3
......................................................................ugacuagacGugcacucaucgcu...................   1     1   Mm3
......................................................................Agacuagaccugcacucaucgcu...................   1     1   Mm3
......................................................................ugacuagaccugcacucaucgcA...................   198   1   Mm3
......................................................................Ngacuagaccugcacucaucgcu...................   3     1   Mm3
......................................................................ugacuagaccugcacuUaucgcu...................   15    1   Mm3
......................................................................ugacuagaccugcacucaucUcu...................   1     1   Mm3
......................................................................ugacuaCaccugcacucaucgcu...................   2     1   Mm3
......................................................................ugacuagaccugcGcucaucgcu...................   2     1   Mm3
......................................................................ugacuagaccugcacucaucgcuU..................   76    1   Mm3
```

Star Mature

uucuguucgugucgcuuauucagauguuuccugcggugagugcacuucuaaucucaugucuacuuauucaugacuagaccugcacucaucgcuggcgacaucuaacccugca			
....ugacuagaccugcacucaucgcAg..................	1	1	Mm3
....gacuagaccugcacucaucgU....................	1	1	Mm3
....gacuagCccugcacucaucgc....................	1	1	Mm3
....gacuagaccugcacucaucgc....................	13	0	Mm3
....gacuagaccugcacucaucgcG...................	2	1	Mm3
....gacuagaccugcacucaucgcu...................	22	0	Mm3
....acuagaccugcacucaucgc....................	1	0	Mm3
....acuagaccugcacucaucgcA...................	2	1	Mm3
....acuagaccugcacucaucgcu...................	6	0	Mm3
....acuagaccugcacucaucgcC...................	1	1	Mm3
....acuagaccugcacucaucgcuU..................	2	1	Mm3
....cuagaccugcacucaucgc....................	1	0	Mm3
..................................ggugagugcacuucuaaucucaug..	7	0	Fm3
....caugacuagaccugcacucauc......................	1	0	Fm3
....Cugacuagaccugcacucaucg.....................	2	1	Fm3
....Cugacuagaccugcacucaucgc....................	18	1	Fm3
....Cugacuagaccugcacucaucgcu...................	19	1	Fm3
....ugacuagaccugcacuca........................	1	0	Fm3
....ugacuagaccugcacucU........................	6	1	Fm3
....ugacuagaccugcacucCu.......................	2	1	Fm3
....ugacuagaccugcacucau.......................	119	0	Fm3
....ugacuagacUugcacucau.......................	1	1	Fm3
....ugacuagaccugcacuUau.......................	1	1	Fm3
....ugacuagaccugcacucUu.......................	1	1	Fm3
....ugacuagaccugcacucauU......................	30	1	Fm3
....ugacuGgaccugcacucauc......................	1	1	Fm3
....ugacuagaccugcGcucauc......................	1	1	Fm3
....ugacuaAaccugcacucauc......................	1	1	Fm3
....ugacuagaccugcacucauA......................	16	1	Fm3
....ugacuagaccugcacucCuc......................	2	1	Fm3
....ugacuagaccugcacuAauc......................	2	1	Fm3
....ugacuagaccGgcacucauc......................	1	1	Fm3
....ugacuagaccugcacucauc......................	340	0	Fm3
....ugacuagaccugcacuUauc......................	1	1	Fm3
....ugacuagaccCgcacucaucg.....................	4	1	Fm3
....ugacuagacUugcacucaucg.....................	2	1	Fm3
....ugGcuagaccugcacucaucg.....................	2	1	Fm3
....ugacuagaccAgcacucaucg.....................	3	1	Fm3
....ugacuagaccugUacucaucg.....................	1	1	Fm3
....ugacuagaccugcacucauUg.....................	1	1	Fm3
....ugacuagaccugcacucCucg.....................	3	1	Fm3
....ugacuagaccugcacuUaucg.....................	1	1	Fm3
....ugacuagaccugcaAucaucg.....................	1	1	Fm3
....ugacuagaccugcacCcaucg.....................	1	1	Fm3
....ugacuagaccugcacucaucg.....................	2082	0	Fm3
....ugacuagaccugcacuAaucg.....................	9	1	Fm3
....uAacuagaccugcacucaucg.....................	3	1	Fm3
....ugacuagaccugcacucaAcg.....................	1	1	Fm3
....ugacuagaUcugcacucaucg.....................	1	1	Fm3
....uUacuagaccugcacucaucg.....................	1	1	Fm3
....ugacuagaccugAacucaucg.....................	1	1	Fm3
....ugacuagaccugcacucGucg.....................	5	1	Fm3
....ugacCagaccugcacucaucg.....................	1	1	Fm3
....ugacuagacAugcacucaucg.....................	1	1	Fm3
....ugacuagaccugcacucaucA.....................	1	1	Fm3
....ugacuagaccugcacuGaucg.....................	1	1	Fm3
....ugacuagaccugcacucUucg.....................	5	1	Fm3
....ugacuagaccGgcacucaucg.....................	2	1	Fm3
....ugacuagGccugcacucaucg.....................	1	1	Fm3
....ugacuagaccugcacucaucU.....................	64	1	Fm3
....ugacuagaccuUcacucaucgc....................	4	1	Fm3
....uAacuagaccugcacucaucgc....................	7	1	Fm3
....ugacuagaccugcacucauGgc....................	2	1	Fm3
....ugacuagaccugcaUucaucgc....................	16	1	Fm3
....ugacuagaccugcUcucaucgc....................	4	1	Fm3
....ugacuagaccugcCcucaucgc....................	1	1	Fm3
....ugacuagacUugcacucaucgc....................	6	1	Fm3
....ugacuagaccugcacucaAcgc....................	1	1	Fm3
....ugacCagaccugcacucaucgc....................	2	1	Fm3

Star **Mature**

```
uucuguucgugucgcuuauucagauguuuccugcggugagugcacuucuaaucucaugucuacuuauucaugacuagaccugcacucaucgcuggcgacaucuaacccugca
......................................................................ugacuagaccugcacCcaucgc....................  1      1  Fm3
......................................................................ugacuagaccugcacucUucgc....................  53     1  Fm3
......................................................................ugacuagaccuAcacucaucgc....................  3      1  Fm3
......................................................................ugaGuagaccugcacucaucgc....................  1      1  Fm3
......................................................................ugacuagaccCgcacucaucgc....................  13     1  Fm3
......................................................................Ggacuagaccugcacucaucgc....................  1      1  Fm3
......................................................................ugacuagaUcugcacucaucgc....................  4      1  Fm3
......................................................................ugacuagaccugcacucaCcgc....................  1      1  Fm3
......................................................................ugacuagaccAgcacucaucgc....................  28     1  Fm3
......................................................................Cgacuagaccugcacucaucgc....................  3      1  Fm3
......................................................................Ngacuagaccugcacucaucgc....................  7      1  Fm3
......................................................................ugacuagaccugcacucaucAc....................  7      1  Fm3
......................................................................ugaUuagaccugcacucaucgc....................  1      1  Fm3
......................................................................ugacuagaccugGacucaucgc....................  1      1  Fm3
......................................................................ugCcuagaccugcacucaucgc....................  1      1  Fm3
......................................................................ugacuNgaccugcacucaucgc....................  2      1  Fm3
......................................................................ugacuaAaccugcacucaucgc....................  2      1  Fm3
......................................................................ugGcuagaccugcacucaucgc....................  8      1  Fm3
......................................................................ugacuagacAugcacucaucgc....................  2      1  Fm3
......................................................................ugacuUgaccugcacucaucgc....................  1      1  Fm3
......................................................................ugacuagaAcugcacucaucgc....................  2      1  Fm3
......................................................................ugacuGgaccugcacucaucgc....................  2      1  Fm3
......................................................................ugacuagaccugcacucaucgA....................  29     1  Fm3
......................................................................ugacuagaccugcacucaucUc....................  1      1  Fm3
......................................................................uCacuagaccugcacucaucgc....................  2      1  Fm3
......................................................................uUacuagaccugcacucaucgc....................  10     1  Fm3
......................................................................ugacuagaccugcGcucaucgc....................  6      1  Fm3
......................................................................Agacuagaccugcacucaucgc....................  3      1  Fm3
......................................................................ugacuagaGcugcacucaucgc....................  1      1  Fm3
......................................................................ugacuagaccugcacucaucgc....................  15612  0  Fm3
......................................................................ugacuagaccugcacucGucgc....................  44     1  Fm3
......................................................................ugacuagaccGgcacucaucgc....................  17     1  Fm3
......................................................................ugacuagaccugcacuGaucgc....................  27     1  Fm3
......................................................................ugacuagaccugUacucaucgc....................  3      1  Fm3
......................................................................ugacuagUccugcacucaucgc....................  1      1  Fm3
......................................................................ugacuCgaccugcacucaucgc....................  1      1  Fm3
......................................................................ugacuagaccugcacucaucgU....................  311    1  Fm3
......................................................................ugacuagaccugcacucCucgc....................  68     1  Fm3
......................................................................ugacuagaccugcacuAaucgc....................  71     1  Fm3
......................................................................ugacuaUaccugcacucaucgc....................  2      1  Fm3
......................................................................ugUcuagaccugcacucaucgc....................  3      1  Fm3
......................................................................ugacuagaccugcacuUaucgc....................  36     1  Fm3
......................................................................ugacuagaccugcacuGaucgcu...................  11     1  Fm3
......................................................................ugacuagaccuAcacucaucgcu...................  2      1  Fm3
......................................................................ugaAuagaccugcacucaucgcu...................  1      1  Fm3
......................................................................ugacuagaccuUcacucaucgcu...................  1      1  Fm3
......................................................................ugacuagaccCgcacucaucgcu...................  1      1  Fm3
......................................................................ugacuagaccugcacucaAcgcu...................  1      1  Fm3
......................................................................ugacuagaccugcacCcaucgcu...................  1      1  Fm3
......................................................................ugacuagaccugcacucaCcgcu...................  1      1  Fm3
......................................................................ugacuagaccugcaUucaucgcu...................  4      1  Fm3
......................................................................ugGcuagaccugcacucaucgcu...................  1      1  Fm3
......................................................................ugacuagaUcugcacucaucgcu...................  1      1  Fm3
......................................................................ugacuagaccugcacucaucgUu...................  35     1  Fm3
......................................................................ugUcuagaccugcacucaucgcu...................  3      1  Fm3
......................................................................uCacuagaccugcacucaucgcu...................  1      1  Fm3
......................................................................ugacuagaccugcacucaucUcu...................  1      1  Fm3
......................................................................ugacuagaccGgcacucaucgcu...................  5      1  Fm3
......................................................................ugacuagaccugcacucUucgcu...................  15     1  Fm3
......................................................................ugacuaUaccugcacucaucgcu...................  1      1  Fm3
......................................................................ugacCagaccugcacucaucgcu...................  1      1  Fm3
......................................................................ugacuagaccugcacucaucCcu...................  1      1  Fm3
......................................................................ugacuagaccugcacuUaucgcu...................  7      1  Fm3
......................................................................ugacuagaccugcacuAaucgcu...................  30     1  Fm3
......................................................................ugacuagaccugcaGucaucgcu...................  1      1  Fm3
......................................................................uAacuagaccugcacucaucgcu...................  4      1  Fm3
......................................................................ugacuagaccugcacucaucgcu...................  5195   0  Fm3
......................................................................ugacuagaccugcacucaucgcC...................  43     1  Fm3
......................................................................ugacuagaccugcacucCucgcu...................  10     1  Fm3
......................................................................ugCcuagaccugcacucaucgcu...................  1      1  Fm3
```

```
                                  Star                                    Mature
uucuguucgugucgcuuauucagauguuuccugcggugagugcacuucuaaucucaugucuacuuauucaugacuagaccugcacucaucgcuggcgacaucuaacccugca
......................................................................ugacuagaccugcGcucaucgcu...................   2    1    Fm3
......................................................................ugacuagaccugcacucauUgcu...................   5    1    Fm3
......................................................................ugacuagaccugcacucaucgcA...................   798  1    Fm3
......................................................................ugacuagacAugcacucaucgcu...................   2    1    Fm3
......................................................................ugacuagaccugUacucaucgcu...................   2    1    Fm3
......................................................................ugacuagaccugGacucaucgcu...................   1    1    Fm3
......................................................................ugacuagaccugcacucGucgcu...................   23   1    Fm3
......................................................................ugacuUgaccugcacucaucgcu...................   1    1    Fm3
......................................................................ugacuagaccAgcacucaucgcu...................   10   1    Fm3
......................................................................ugacuagaccugcacucaucgcG...................   10   1    Fm3
......................................................................Ngacuagaccugcacucaucgcu...................   1    1    Fm3
......................................................................Cgacuagaccugcacucaucgcu...................   2    1    Fm3
......................................................................ugacuagaccugcacucaucgcuU..................   177  1    Fm3
.......................................................................gacuagaccuCcacucaucg.....................   1    1    Fm3
.......................................................................gacuagaccugcacucaucg.....................   9    0    Fm3
.......................................................................gacuagaccuUcacucaucgc....................   1    1    Fm3
.......................................................................gacuagaccugcacCcaucgc....................   1    1    Fm3
.......................................................................gacuagaccugcacucaucgU....................   3    1    Fm3
.......................................................................gacuagaccugcacucaucgc....................   42   0    Fm3
.......................................................................gacuagaccuCcacucaucgc....................   1    1    Fm3
.......................................................................gacuaCaccugcacucaucgc....................   1    1    Fm3
.......................................................................gacuagaccugcacucaucgcu...................   19   0    Fm3
.......................................................................gacuagaccugcaGucaucgcu...................   1    1    Fm3
.......................................................................gacuagaccuAcacucaucgcu...................   1    1    Fm3
.......................................................................gacuagaccugcacucaucgcA...................   1    1    Fm3
.......................................................................gacuagaccugcacucaucgcuU..................   2    1    Fm3
........................................................................acuagaccugcacucaucgcG...................   3    1    Fm3
........................................................................acuagaccugcacucaucgcu...................   9    0    Fm3
........................................................................acuagaccugcacucaucgcA...................   2    1    Fm3
........................................................................acuagaccugcacucaucgcC...................   3    1    Fm3
........................................................................acuagaccugcacucaucgcuU..................   4    1    Fm3

..................................ggugagugcGcuucuaaucucaug......................................................   1    1    Mm2
..................................ggugagugcacuucuaaGcucaug......................................................   1    1    Mm2
..................................ggugagugcacuucuaaucucaug......................................................   41   0    Mm2
..........................................................ucuacuuauucaugacuagaccu...............................   1    0    Mm2
..........................................................ucuacuuauucaugacuagaccugc.............................   5    0    Mm2
..........................................................ucuacuuauucaugacuagaccugcacucaucgc....................   1    0    Mm2
...................................................................ucaugacuagaccugcacucaucgc....................   2    0    Mm2
....................................................................caugacuagaccugcacucaucgc....................   1    0    Mm2
.....................................................................Cugacuagaccugcacucaucgc....................   5    1    Mm2
.....................................................................Cugacuagaccugcacucaucgcu...................   1    1    Mm2
......................................................................ugacuagaccugcacucU........................   3    1    Mm2
......................................................................ugacuagaccugcacuca........................   1    0    Mm2
......................................................................ugacuagaccugcacucau.......................   5    0    Mm2
......................................................................ugacuagaccugcacucauU......................   4    1    Mm2
......................................................................ugacuagaccugcacuUauc......................   1    1    Mm2
......................................................................ugacuagaccugcacucauc......................   42   0    Mm2
......................................................................ugacuagaccugcacucaucU.....................   4    1    Mm2
......................................................................ugacuagaccugcGcucaucg.....................   1    1    Mm2
......................................................................ugacuagacAugcacucaucg.....................   1    1    Mm2
......................................................................uNacuagaccugcacucaucg.....................   1    1    Mm2
......................................................................ugacuagaccugcacucCucg.....................   2    1    Mm2
......................................................................ugacuagaccugcacucaucg.....................   206  0    Mm2
......................................................................ugacuagaccugcacucGucg.....................   2    1    Mm2
......................................................................ugacuagaccCgcacucaucgc....................   5    1    Mm2
......................................................................ugacuagaccugcacucaucgU....................   42   1    Mm2
......................................................................Agacuagaccugcacucaucgc....................   1    1    Mm2
......................................................................ugacuagaccugcGcucaucgc....................   4    1    Mm2
......................................................................ugacuagaUcugcacucaucgc....................   6    1    Mm2
......................................................................ugacuUgaccugcacucaucgc....................   1    1    Mm2
......................................................................ugacuagaccugcacucGucgc....................   53   1    Mm2
......................................................................ugacuagaccuCcacucaucgc....................   1    1    Mm2
......................................................................uCacuagaccugcacucaucgc....................   2    1    Mm2
......................................................................Cgacuagaccugcacucaucgc....................   2    1    Mm2
......................................................................ugacuagaccugcacucUucgc....................   16   1    Mm2
......................................................................ugacuagaccugcacucaucAc....................   3    1    Mm2
......................................................................ugCcuagaccugcacucaucgc....................   1    1    Mm2
......................................................................ugacuagaccugcacucauUgc....................   7    1    Mm2
......................................................................ugacuagaccGgcacucaucgc....................   9    1    Mm2
```

```
                                Star                                Mature
uucuguucgugucgcuuauucagauguuucugcggugagugcacuucuaaucucaugucuacuuauucaugacuagaccugcacucaucgcuggcgacaucuaacccugca
.....................................................................ugacuagaAcugcacucaucgc....................    1     1    Mm2
.....................................................................ugGcuagaccugcacucaucgc....................    5     1    Mm2
.....................................................................ugacuagaccugcacucaucgA....................    14    1    Mm2
.....................................................................ugacuagaccugcacCcaucgc....................    1     1    Mm2
.....................................................................ugacuagaccuUcacucaucgc....................    2     1    Mm2
.....................................................................ugacuagaccugcacuUaucgc....................    17    1    Mm2
.....................................................................ugacuagaccugcacucaucgc....................    7727  0    Mm2
.....................................................................ugUcuagaccugcacucaucgc....................    2     1    Mm2
.....................................................................uAacuagaccugcacucaucgc....................    1     1    Mm2
.....................................................................ugacuagaccugcacucaucgG....................    9     1    Mm2
.....................................................................ugacuagaccugAacucaucgc....................    1     1    Mm2
.....................................................................ugacuagaccugcaUucaucgc....................    2     1    Mm2
.....................................................................ugacuagaccAgcacucaucgc....................    13    1    Mm2
.....................................................................ugacuaUaccugcacucaucgc....................    3     1    Mm2
.....................................................................Ngacuagaccugcacucaucgc....................    7     1    Mm2
.....................................................................ugacuGgaccugcacucaucgc....................    4     1    Mm2
.....................................................................ugacuagaccugcacucaAcgc....................    3     1    Mm2
.....................................................................ugacuagaccugcacuGaucgc....................    7     1    Mm2
.....................................................................ugacuagaGcugcacucaucgc....................    1     1    Mm2
.....................................................................ugacuagaccugUacucaucgc....................    4     1    Mm2
.....................................................................ugacuaAaccugcacucaucgc....................    2     1    Mm2
.....................................................................ugacuagacUugcacucaucgc....................    7     1    Mm2
.....................................................................ugacuagaccugcacuAaucgc....................    29    1    Mm2
.....................................................................ugacAagaccugcacucaucgc....................    1     1    Mm2
.....................................................................ugaAuagaccugcacucaucgc....................    1     1    Mm2
.....................................................................uUacuagaccugcacucaucgc....................    1     1    Mm2
.....................................................................ugacuagCccugcacucaucgc....................    1     1    Mm2
.....................................................................ugacuagacAugcacucaucgc....................    1     1    Mm2
.....................................................................ugacuagaccugcacucCucgc....................    34    1    Mm2
.....................................................................ugacuagaccugcacuAaucgcu...................    5     1    Mm2
.....................................................................ugacuagaccugcacuUaucgcu...................    1     1    Mm2
.....................................................................ugacuagaccGgcacucaucgcu...................    5     1    Mm2
.....................................................................ugacuagaccugcacucUucgcu...................    2     1    Mm2
.....................................................................ugacuagaccugcacucaucgcu...................    780   0    Mm2
.....................................................................ugacuagacUugcacucaucgcu...................    1     1    Mm2
.....................................................................ugacuagaccugcacucaucgcA...................    88    1    Mm2
.....................................................................ugacuagaccAgcacucaucgcu...................    4     1    Mm2
.....................................................................Ngacuagaccugcacucaucgcu...................    1     1    Mm2
.....................................................................uCacuagaccugcacucaucgcu...................    1     1    Mm2
.....................................................................Cgacuagaccugcacucaucgcu...................    1     1    Mm2
.....................................................................ugacuagaccugcacuGaucgcu...................    1     1    Mm2
.....................................................................ugacuagaccugcacucaucgcC...................    11    1    Mm2
.....................................................................ugacGagaccugcacucaucgcu...................    1     1    Mm2
.....................................................................ugUcuagaccugcacucaucgcu...................    1     1    Mm2
.....................................................................ugacuagaccugcacucGucgcu...................    1     1    Mm2
.....................................................................uAacuagaccugcacucaucgcu...................    1     1    Mm2
.....................................................................ugacuagaccugcacucaucgcG...................    73    1    Mm2
.....................................................................ugacuagaccCgcacucaucgcu...................    1     1    Mm2
.....................................................................ugaAuagaccugcacucaucgcu...................    1     1    Mm2
.....................................................................ugacuagaccugcacucaucgUu...................    5     1    Mm2
.....................................................................ugacuagaccugcaUucaucgcu...................    1     1    Mm2
.....................................................................ugacuagaccugUacucaucgcu...................    1     1    Mm2
.....................................................................ugacuagaccugcacucaucgcug..................    1     0    Mm2
.....................................................................ugacuagaccugcacucaucgcuU..................    15    1    Mm2
.....................................................................ugacuagaccugcacucaucgcuA..................    1     1    Mm2
......................................................................gacuagaccugcacucaucgU....................    2     1    Mm2
......................................................................gacuagaccugcacucaucgc....................    7     0    Mm2
......................................................................gacuagGccugcacucaucgcu...................    1     1    Mm2
......................................................................gacuagaccugcacucaucgcG...................    1     1    Mm2
.......................................................................acuagaccugcacucauc......................    1     0    Mm2
.......................................................................acuagaccugcacucaucgc....................    2     0    Mm2
.......................................................................acuagaccugcacucaucgcG...................    1     1    Mm2
.......................................................................acuagaccugcacucaucgcC...................    1     1    Mm2
.......................................................................acuagaccugcacucaucgcu...................    5     0    Mm2
.......................................................................acuagaccugcGcucaucgcu...................    1     1    Mm2
.......................................................................acuagaccugcacucaucgcA...................    2     1    Mm2
.......................................................................acuagaccugcacucaucgcuU..................    8     1    Mm2

....................................................................Cugacuagaccugcacucaucgc....................    21    1    Mm1
....................................................................Cugacuagaccugcacucaucgcu...................    46    1    Mm1
```

Star Mature

uucuguucgugucgcuuauucagauguuuccugcggugagugcacuucuaaucucaugucuacuuauucaugacuagaccugcacucaucgcuggcgacaucuaacccugca			
....ugacuagaccugcacuca........................	20	0	Mm1
....ugacuagaccugcacucGuc......................	1	1	Mm1
....ugacuagaccugcacucCuc......................	1	1	Mm1
....ugacuagaccGgcacucauc......................	1	1	Mm1
....ugacuagaccugcacucauc......................	107	0	Mm1
....ugacuagaccugcacucaucg.....................	290	0	Mm1
....ugacuagaccugcacucGucg.....................	1	1	Mm1
....ugacuaUaccugcacucaucg.....................	1	1	Mm1
....ugacuagaccugcacuUaucg.....................	1	1	Mm1
....ugacuagaccugcacucCucg.....................	2	1	Mm1
....ugacuagaccugcGcucaucg.....................	1	1	Mm1
....ugacuagaAcugcacucaucg.....................	1	1	Mm1
....ugUcuagaccugcacucaucgc....................	2	1	Mm1
....ugacuagaccugcacucaucUc....................	2	1	Mm1
....ugacuagaccugcacuUaucgc....................	5	1	Mm1
....ugacuagaccugcacucCucgc....................	18	1	Mm1
....ugacuagaccugcacuAaucgc....................	14	1	Mm1
....ugacuagaccugcUcucaucgc....................	2	1	Mm1
....ugacuagaccugcacuGaucgc....................	12	1	Mm1
....uAacuagaccugcacucaucgc....................	4	1	Mm1
....ugacuagaUcugcacucaucgc....................	3	1	Mm1
....ugacuagaccugcacucauUgc....................	7	1	Mm1
....ugGcuagaccugcacucaucgc....................	2	1	Mm1
....ugacuagacUugcacucaucgc....................	1	1	Mm1
....ugaUuagaccugcacucaucgc....................	1	1	Mm1
....uUacuagaccugcacucaucgc....................	1	1	Mm1
....ugacuagaccugcacucGucgc....................	16	1	Mm1
....ugacuagaccugcaAucaucgc....................	1	1	Mm1
....ugacuagaccugcacucaucgc....................	4870	0	Mm1
....ugacuagaccugUacucaucgc....................	2	1	Mm1
....ugacuagaccGgcacucaucgc....................	6	1	Mm1
....ugacuagaccugcacucUucgc....................	13	1	Mm1
....ugacuNgaccugcacucaucgc....................	1	1	Mm1
....ugacuaUaccugcacucaucgc....................	2	1	Mm1
....ugacuagaccugcacucaucAc....................	1	1	Mm1
....Ngacuagaccugcacucaucgc....................	2	1	Mm1
....ugacuagaccugcacucaucgU....................	22	1	Mm1
....ugacuagaGcugcacucaucgc....................	1	1	Mm1
....ugacuagaccCgcacucaucgc....................	4	1	Mm1
....ugacuagaccAgcacucaucgc....................	7	1	Mm1
....ugacuagaccGgcacucaucgcu...................	13	1	Mm1
....ugacuagaccuAcacucaucgcu...................	1	1	Mm1
....ugacuaNaccugcacucaucgcu...................	1	1	Mm1
....ugacuagaUcugcacucaucgcu...................	1	1	Mm1
....ugacuagaccugcacAcaucgcu...................	1	1	Mm1
....ugacuagaccugcacucCucgcu...................	16	1	Mm1
....ugacuagaccugAacucaucgcu...................	1	1	Mm1
....ugacuagaccugcacucaucgcA...................	161	1	Mm1
....ugacuagaccugcacucGucgcu...................	21	1	Mm1
....ugCcuagaccugcacucaucgcu...................	1	1	Mm1
....ugacuagaccugcacucaucgAu...................	1	1	Mm1
....ugacuagaccugcacucaucgcu...................	5768	0	Mm1
....ugacuagaccugUacucaucgcu...................	9	1	Mm1
....ugacuagaccugcacuGaucgcu...................	11	1	Mm1
....Cgacuagaccugcacucaucgcu...................	1	1	Mm1
....uUacuagaccugcacucaucgcu...................	2	1	Mm1
....ugacuagaccCgcacucaucgcu...................	9	1	Mm1
....ugacuagaccugcacucaucUcu...................	1	1	Mm1
....ugacuagaccugcacucaucgUu...................	15	1	Mm1
....ugacuagaAcugcacucaucgcu...................	1	1	Mm1
....ugacuagaccugcacucauUgcu...................	4	1	Mm1
....ugacuagaccugcacuAaucgcu...................	23	1	Mm1
....ugacuagUccugcacucaucgcu...................	2	1	Mm1
....ugacAagaccugcacucaucgcu...................	1	1	Mm1
....ugacuagaccAgcacucaucgcu...................	9	1	Mm1
....ugacuaCaccugcacucaucgcu...................	1	1	Mm1
....ugacuagaccugcacuUaucgcu...................	9	1	Mm1
....ugacuagaccugcacucaucgcG...................	67	1	Mm1
....ugGcuagaccugcacucaucgcu...................	2	1	Mm1
....ugacuagaccugcacucaCcgcu...................	1	1	Mm1

```
                                  Star                                  Mature
uucuguucgugucgcuuauucagauguuuccugcggugagugcacuucuaaucucaugucuacuuauucaugacuagaccugcacucaucgcuggcgacaucuaacccugca
......................................................................ugacuUgaccugcacucaucgcu...................   1     1   Mm1
......................................................................ugacuagaccugcaUucaucgcu...................   2     1   Mm1
......................................................................ugacuagaccugcacucaucgcC...................   27    1   Mm1
......................................................................Ngacuagaccugcacucaucgcu...................   3     1   Mm1
......................................................................ugacuagacUugcacucaucgcu...................   1     1   Mm1
......................................................................uAacuagaccugcacucaucgcu...................   1     1   Mm1
......................................................................Agacuagaccugcacucaucgcu...................   1     1   Mm1
......................................................................ugacuagaccugcacucUucgcu...................   16    1   Mm1
......................................................................ugacCagaccugcacucaucgcu...................   1     1   Mm1
......................................................................ugacuagaccuCcacucaucgcu...................   1     1   Mm1
......................................................................ugacuaUaccugcacucaucgcu...................   2     1   Mm1
......................................................................ugacuagaccugcacCcaucgcu...................   1     1   Mm1
......................................................................ugacuagaccuUcacucaucgcu...................   1     1   Mm1
......................................................................ugacuagGccugcacucaucgcu...................   1     1   Mm1
......................................................................ugUcuagaccugcacucaucgcu...................   1     1   Mm1
......................................................................ugacuagacAugcacucaucgcu...................   1     1   Mm1
......................................................................ugacuagaccugcGcucaucgcu...................   2     1   Mm1
......................................................................ugacuagaccugcacucaucgcuU..................   24    1   Mm1
......................................................................ugacuagaccugcacucaucgcCg..................   2     1   Mm1
.......................................................................gacuagaAcugcacucaucgc....................   1     1   Mm1
.......................................................................gacuagaccugcacucaucgc....................   4     0   Mm1
.......................................................................gacuagaccugcacucaucgcu...................   7     0   Mm1
.........................................................................cuagaccugAacucaucgc....................   1     1   Mm1
.........................................................................cuagaccugcacucaucgc....................   4     0   Mm1

..................................ggugagugcacuucuaaGcucaug......................................................   1     1   Fm1
..................................ggugagugcacuucuaaucucaug......................................................   22    0   Fm1
......................................................................ugacuagaccugcacucC........................   1     1   Fm1
......................................................................ugacuagaccugcacuca........................   1     0   Fm1
......................................................................ugacuagaccugcacucau.......................   18    0   Fm1
......................................................................ugacuagaccugcacucauU......................   4     1   Fm1
......................................................................ugacuagaccugcacucauc......................   72    0   Fm1
......................................................................ugacuagaccugUacucaucg.....................   3     1   Fm1
......................................................................ugacuagaccugcacucCucg.....................   1     1   Fm1
......................................................................ugacuagaccugcacucaucg.....................   260   0   Fm1
......................................................................ugacuagaccugcacuAaucg.....................   1     1   Fm1
......................................................................ugacuagaccugcacucaucU.....................   2     1   Fm1
......................................................................ugacuagaccCgcacucaucg.....................   1     1   Fm1
......................................................................ugacuagUccugcacucaucgc....................   1     1   Fm1
......................................................................ugacuagaccugcGcucaucgc....................   3     1   Fm1
......................................................................uAacuagaccugcacucaucgc....................   2     1   Fm1
......................................................................ugacuagaccugcacuAaucgc....................   34    1   Fm1
......................................................................ugacuagaccugcacucaucgc....................   7936  0   Fm1
......................................................................ugacuUgaccugcacucaucgc....................   1     1   Fm1
......................................................................ugacuagaccugcacCcaucgc....................   1     1   Fm1
......................................................................ugacuagaccugcacucUucgc....................   13    1   Fm1
......................................................................ugacuagaccugcacucaucUc....................   1     1   Fm1
......................................................................ugacuagaccugAacucaucgc....................   3     1   Fm1
......................................................................ugacuagaccCgcacucaucgc....................   9     1   Fm1
......................................................................ugaAuagaccugcacucaucgc....................   2     1   Fm1
......................................................................ugCcuagaccugcacucaucgc....................   2     1   Fm1
......................................................................ugacuagaccugcacucauUgc....................   1     1   Fm1
......................................................................ugGcuagaccugcacucaucgc....................   5     1   Fm1
......................................................................ugacuaNaccugcacucaucgc....................   1     1   Fm1
......................................................................uUacuagaccugcacucaucgc....................   3     1   Fm1
......................................................................Agacuagaccugcacucaucgc....................   1     1   Fm1
......................................................................ugacuagaccugUacucaucgc....................   3     1   Fm1
......................................................................ugacuGgaccugcacucaucgc....................   3     1   Fm1
......................................................................ugacuagaccugcacucaucgA....................   5     1   Fm1
......................................................................ugacuagaccugcacucGucgc....................   33    1   Fm1
......................................................................ugacuagCccugcacucaucgc....................   1     1   Fm1
......................................................................Ggacuagaccugcacucaucgc....................   1     1   Fm1
......................................................................ugacuagacAugcacucaucgc....................   1     1   Fm1
......................................................................ugacuagaccugcacucaucgG....................   12    1   Fm1
......................................................................ugacuagaccugcacucauAgc....................   1     1   Fm1
......................................................................ugacuagacUugcacucaucgc....................   7     1   Fm1
......................................................................Cgacuagaccugcacucaucgc....................   2     1   Fm1
......................................................................ugacuagaccugcacuGaucgc....................   9     1   Fm1
......................................................................ugacuagaUcugcacucaucgc....................   7     1   Fm1
......................................................................ugacuagaccugcacuUaucgc....................   23    1   Fm1
```

Star Mature

uucuguucgugucgcuuauucagauguuuccugcggugagugcacuucuaaucucaugucuacuuauucaugacuagaccugcacucaucgcuggcgacaucuaacccugca			
....ugaUuagaccugcacucaucgc....................	1	1	Fm1
....ugacuagaccugcacucaucAc....................	6	1	Fm1
....ugacuagaccugcacucCucgc....................	31	1	Fm1
....ugacuaUaccugcacucaucgc....................	3	1	Fm1
....ugacuagaccGgcacucaucgc....................	9	1	Fm1
....ugacuagaccugcacucaucgU....................	58	1	Fm1
....ugacuagaccAgcacucaucgc....................	11	1	Fm1
....ugacuagaccAgcacucaucgcu...................	3	1	Fm1
....ugacuagacUugcacucaucgcu...................	3	1	Fm1
....ugacuagaccCgcacucaucgcu...................	2	1	Fm1
....ugacuNgaccugcacucaucgcu...................	1	1	Fm1
....ugacuagaccugcacucaucgcG...................	80	1	Fm1
....ugacuagaccugcacucGucgcu...................	1	1	Fm1
....Ngacuagaccugcacucaucgcu...................	1	1	Fm1
....ugacuagaccugcacucaucgAu...................	1	1	Fm1
....ugacuagaccugcacuUaucgcu...................	1	1	Fm1
....ugacuagaccugcaGucaucgcu...................	1	1	Fm1
....ugacuagaccugcacucaucgUu...................	3	1	Fm1
....uNacuagaccugcacucaucgcu...................	1	1	Fm1
....Cgacuagaccugcacucaucgcu...................	1	1	Fm1
....ugacuagaccugcacuAaucgcu...................	5	1	Fm1
....ugacuagaccugcacucauUgcu...................	4	1	Fm1
....ugacuagaccugcacucaucgcA...................	86	1	Fm1
....ugacuagaccugcacucaucgcC...................	10	1	Fm1
....ugacuagaccugcacucCucgcu...................	1	1	Fm1
....ugacuagaccugcacuGaucgcu...................	1	1	Fm1
....ugacuagaccugcacucaucAcu...................	1	1	Fm1
....ugacuagaccugcacucaucgcu...................	873	0	Fm1
....ugGcuagaccugcacucaucgcu...................	1	1	Fm1
....ugacuagaccugcacucaucgcuU..................	95	1	Fm1
....gacuagaccugcacucaucgc....................	1	0	Fm1
....gacuagaccuAcacucaucgc....................	1	1	Fm1
....acuagaccugcacucaucgc....................	3	0	Fm1
..................................ggugagugcacuucuaaucucaug..	26	0	Fm2
..................................ggugagugcaUuucuaaucucaug..	1	1	Fm2
....augacuagaccugcacucaucgcu...................	8	0	Fm2
....augacuagaccugcacucaucgcG...................	2	1	Fm2
....ugacuagaccugcacucauc......................	5	0	Fm2
....ugacuagaccugcacucGucg.....................	1	1	Fm2
....ugacuagaccugcacucaucg.....................	42	0	Fm2
....ugUcuagaccugcacucaucg.....................	1	1	Fm2
....ugacuagaccGgcacucaucgc....................	1	1	Fm2
....ugacuagaccCgcacucaucgc....................	1	1	Fm2
....ugacuagaccugcacucaucgA....................	1	1	Fm2
....ugacuNgaccugcacucaucgc....................	1	1	Fm2
....ugacuagaccuAcacucaucgc....................	1	1	Fm2
....ugacuagaccAgcacucaucgc....................	1	1	Fm2
....ugGcuagaccugcacucaucgc....................	1	1	Fm2
....ugacuagaccugcacucGucgc....................	4	1	Fm2
....ugacuagaccugAacucaucgc....................	1	1	Fm2
....ugacuGgaccugcacucaucgc....................	1	1	Fm2
....ugacuagaUcugcacucaucgc....................	2	1	Fm2
....ugacuagaccugcacucaucgG....................	1	1	Fm2
....Ngacuagaccugcacucaucgc....................	1	1	Fm2
....ugacuagaccugcacucaucgc....................	1298	0	Fm2
....ugacuagaccugcacuGaucgc....................	1	1	Fm2
....ugacuagaccugcacuAaucgc....................	7	1	Fm2
....ugacuagaccugcacucUucgc....................	2	1	Fm2
....ugacuagaccugcacucaucAc....................	1	1	Fm2
....ugacuagaccugcacuUaucgc....................	2	1	Fm2
....ugacuagaccugcacucaucgU....................	12	1	Fm2
....ugacuagaccugcacucCucgc....................	3	1	Fm2
....ugacuagaccugcacuGaucgcu...................	1	1	Fm2
....ugacuagaccugcacuAaucgcu...................	2	1	Fm2
....ugacuagaccugcacucaucgcG...................	47	1	Fm2
....ugacuagaccAgcacucaucgcu...................	3	1	Fm2
....ugacuagaccugcacucUucgcu...................	2	1	Fm2
....ugacuagaccugcacucaucgcu...................	485	0	Fm2
....ugUcuagaccugcacucaucgcu...................	1	1	Fm2

Star **Mature**

```
uucuguucgugucgcuuauucagauguuuccugcggugagugcacuucuaaucucaugucuacuuauucaugacuagaccugcacucaucgcuggcgacaucuaacccugca
......................................................................ugacuagaccuCcacucaucgcu...................  1   1   Fm2
......................................................................ugacuNgaccugcacucaucgcu...................  1   1   Fm2
......................................................................ugacuagaccugcacucauUgcu...................  2   1   Fm2
......................................................................ugacuagaccugcaUucaucgcu...................  1   1   Fm2
......................................................................ugacuagaccuAcacucaucgcu...................  1   1   Fm2
......................................................................uAacuagaccugcacucaucgcu...................  1   1   Fm2
......................................................................ugacuagaccugcacucaucgcA...................  28  1   Fm2
......................................................................ugacuagacUugcacucaucgcu...................  1   1   Fm2
.......................................................................gacuagaccugcacucaucgc....................  3   0   Fm2
.......................................................................gacuagaUcugcacucaucgc....................  1   1   Fm2
........................................................................acuagaccugcacucaucgcu...................  1   0   Fm2
```
